# A 4-port flexible MIMO antenna with isolation enhancement for wireless IoT applications

**DOI:** 10.1016/j.heliyon.2024.e32216

**Published:** 2024-05-31

**Authors:** Uktam Fakhriddinovich Azimov, Anees Abbas, Seong-Wook Park, Niamat Hussain, Nam Kim

**Affiliations:** a Department of Information and Communication, Chungbuk National University, Cheongju, 28644, Republic of Korea; b Department of Department of Intelligent Mechatronics Engineering, Sejong University, Seoul, 05006, Republic of Korea

**Keywords:** Flexible MIMO monopole antenna, ISM band, WLAN band, IoT band antenna

## Abstract

This paper presents a conformal, miniaturized, and geometrically simple monopole antenna designed for Vehicle–to–Everything (V2X) communications. The antenna consists of a flexible substrate, radiating patch, ground, and metallic stubs. Meandered lines are added to the U-shaped radiator to achieve the required bandwidth of the antenna. The antenna has |S_11_|< −10 dB magnitude from 5.06 to 7.24 GHz, attaining a peak low magnitude of–68 dB. The antenna is configured into a 4-port Multiple–Input–Multiple–Output (MIMO) setup to minimize the mutual coupling between its elements. The proposed flexible MIMO antenna offers bandwidth from 5.37 to 7.34 GHz and a peak moderate gain of 4.63 dBi with omnidirectional stable radiation patterns. To improve the mutual coupling, two hollow concentric circular structures, in combination with a pair of stub networks are integrated between the elements of the MIMO system. The transmission coefficient and surface current analysis confirm the effectiveness of the decoupling structure. The presented MIMO antenna is characterized by high isolation, a low envelope correlation coefficient (ECC), and high diversity gain, suitable for V2X MIMO communication scenarios.

## 1 Introduction

A trend in the exponential increase of the number of wireless devices has been observed in the current era. With the advent of pervasive computing, the demand for Internet–of–Things (IoT) devices will also cause an amelioration in this drift. By the end of 2024, it is projected that the number of IoT devices will quadruple the amount of population in the world [1,2]. The ubiquitous use of IoT devices finds its application in smart vehicles, factories, intelligent health monitoring equipment, and communication links for seamless high-rate transfer for connecting people [3,4]. IoT is an intersection between humans, machines, and internet connectivity. With the widespread internet ubiquity, it is a mode of the most common communication. IoT devices have exploited this significant advantage of the internet and allowed a cost-effective methodology for digital communication. The key idea is to allow low devices to be connected to the internet for digital communication and data transfer. Hence, IoT devices are readily found in agricultural, industrial, biomedical, vehicular, smart cities, and educational setups [[5], [6], [7]].

The Multiple-Input-Multiple-Output (MIMO) technology is a promising technology that can fulfill the requirements of high-rate transmission rates, improved communication quality, low path fading, and multiple channels for communication [[8], [9], [10]]. To achieve design miniaturization, the MIMO systems should have antennas placed closer to each other. However, this comes with an increase in the channel capacity loss and mutual coupling between the radiating elements [[11], [12], [13]]. Thus, keeping the low spacing between the antenna elements while reducing the mutual coupling between the radiating elements is a design challenge due to which innovative decoupling structures are incorporated in the design. Numerous techniques have been explored in the literature for the overall mutual coupling reduction between the elements, while keeping the minimum spacing between the elements [[14], [15], [16], [17], [18]].

In [15], a graphene-based frequency selective surface is utilized for mutual coupling reduction among antenna components. The plasmonic nano-antenna cluster has a decoupling structure offering a stopband from 1.1 to 1.7 THz. The decoupling structure offers a 15 dB isolation improvement in the coupling between the elements. Furthermore, the presented antenna has a lower-than–0.01 envelope correlation coefficient, applicable for practical applications. In Ref. [16], and [19], a ground stub and an Electromagnetic Bandgap (EBG) structure are utilized for mutual coupling reduction. The ground plane is partial with the radiating elements sharing the same ground plane. Thus, mutual coupling reduction techniques include modifying the ground plane along with the utilization of EBG for the same purpose. In Ref. [17], a MIMO system for V2X communication, operating at 5.9 GHz is presented. The radiating element has a circular polarization, which provides low cross-polarization isolation. The MIMO system uses a parasitic patch between the radiating elements and introduces circular slots in the ground plane. Extensive parametric analysis is used to optimize the dimensions of slots. The MIMO antenna offers a high isolation of 34 dB with a net radiation efficiency of 94 % [20]. The peak gain of the presented design is 7.68 dB, suitable for high–gain applications. However, the presented antenna does not offer conformance and is geometrically large as compared to the design presented in this article [18]. provides an extensive review of the number of methods and techniques which are currently employed for the miniaturization of the MIMO element along with the reduction in the mutual coupling between the elements. In Ref. [21], a four-port, decagon–like antenna is presented in the literature. The presented antenna is an easy-to-fabricate structure and offers flexibility, valuable for flexible electronics. The MIMO antenna has overall dimensions of 45 × 38 × 0.2 mm3, with an overall radiation efficiency of 88 percent. However, the presented antenna has larger dimensions and ECC values of <0.04. In Ref. [22], a compact 2 × 2-element MIMO antenna is presented with a unique parasitic patch between the radiating structures. The antenna offers a moderate gain of 4.3 dBi. The presented antenna is geometrically large and does not offer flexibility for conformable devices. In Ref. [23], a moderate gain antenna is presented with dimensions of 38 × 38 × 1.524 mm3. The design has a bandwidth coverage of 5.3–6.7 GHz with a peak radiation efficiency of 70 percent. The design utilizes a uniquely defective ground structure for the optimization at the target frequency. The design efficiency and relatively less radiation efficiency and has a geometrically large structure. Moreover, in Ref. [24], a geometrically small, stiff, and low-gain antenna is presented in the literature. The design is efficient in spacing between the elements as the edge–to–edge spacing between the propagation elements is 0.076 λ0. This antenna has less bandwidth coverage as compared to the presented design and also is geometrically stiff, not suitable for flexible electronic applications. In Ref. [25], a two-element fractal antenna is presented for WiFi applications at 5.8 GHz. The antenna has dimensions of 72.7 × 36.65 × 1.6 mm^3^ fabricated on a cheap FR–4 substrate and is suitable for mass production. However, the antenna is geometrically large, has a bandwidth of 540 MHz, and is rigid.

In [26], a circularly polarized, three–dimensional antenna is presented with a good isolation figure of 17 dB. The three-dimensional prototype has a bandwidth coverage of 5.15–6.12 GHz of frequency, suitable for vehicular applications. The antenna offers excellent directional characteristics, along with a good electrical field distribution pattern. However, the antenna is a three–dimensional inflexible structure, which may or may not be a good feature, depending upon the application. In the same way, a structurally large two–port MIMO antenna is presented in Ref. [27] with an isolation of 12 dB for mobile handset applications. The antenna uses a defected ground structure, along with a plane inverted F-shaped structure for achieving |S_11_| < −10 dB magnitude from 5.1 to 6 GHz. The presented design is inelastic and not suitable for applications where flexibility is a core requirement. Finally, a two-element ultrawide band literature presents a MIMO antenna. The use of a partial ground plane in this design with a hollow circle as the main propagation element. The proposed design has directional radiation characteristics due to the use of the partial ground plane. The antenna has an efficiency of more than eighty percent throughout the operational region of the frequency spectrum. However, the MIMO antenna does not have conformity, thereby limiting its operational characteristics [28].

The widespread use of IoT applications asks for high data rate antenna systems that are compatible with flexible electronic systems. Designing a flexible antenna for vehicle applications has enormous potential for allowing seamless connectivity in emerging vehicle-to-everything (V2X) communication systems. The proposed antenna has the potential to improve autonomous driving, smart traffic management, and real-time vehicle communication, all of which will define the future of transportation.

This paper presents a flexible, miniaturized, and low mutual coupling MIMO antenna for applications like vehicular IoT. The improvement in bandwidth and radiation characteristics is achieved by an extensive parametric analysis over various dimensions of the radiation elements. Furthermore, to improve the mutual coupling parameter, a unique network of stubs is introduced between the perpendicularly placed antenna elements. The design process, parametric analysis, comparison with the state–of–the–artworks, and important performance metrics of the proposed MIMO antenna are all thoroughly discussed in the later sections. Section 2 discusses the design aspects of the proposed antenna, whereas section 3 discusses the comparison with the state–of–the–artworks. Furthermore, Section 4 concludes the entire article.

## 2 Antenna design

### 2.1 Single element antenna geometry

**Fig. 1:**
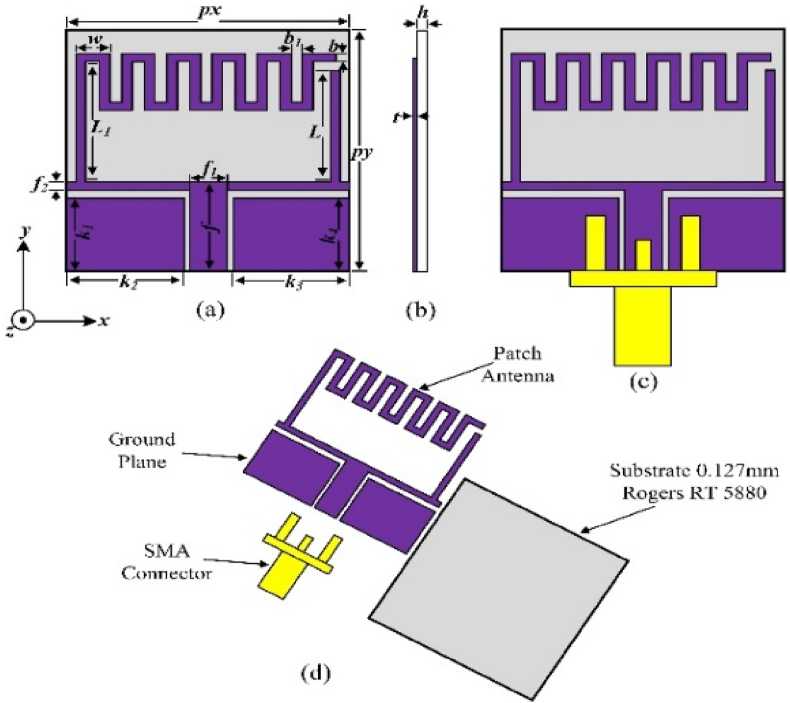
The flexible monopole antenna's geometry (a) front view (b) side view (c) antenna with connector and (d) Exploded View.

The geometry of the proposed conformal, miniaturized, low correlation coefficient single unit antenna is presented in Fig. 1(a–d). The antenna is etched on the top side of commercially available Rogers 5880 laminate, having a dielectric constant of 2.20 and loss tangent of 0.0009. Initially, a Coplanar waveguide (CPW) fed rectangular patch is designed at the resonance frequency of 5.8 GHz. The antenna's resonance frequency and effective dielectric constant values can be numerically obtained from Equations (1), (2), respectively [29,30].

(1) $$f_{r}=\frac{c}{{4L}_{0}\sqrt{\varepsilon_{eff}}}$$

The effective dielectric constant can be numerically calculated by the following equation.

(2) $$\varepsilon_{eff}=\frac{\varepsilon_{r}+1}{2}+\frac{\varepsilon_{r}+1}{2}\{{\left(1+12\frac{h}{\omega }\right)}^{-0.5}+0.04\;{\left(1+12\frac{h}{\omega }\right)}^{2}\}$$

The length and width of the radiation patch can be calculated using the following equations (3), (4), (5).

(3) $$L=l_{eff}-2\Delta l$$

(4) Ls=6h+l

(5) Ws=6h+w

A U-shaped radiator is the core element of the traditional monopole patch antenna, and the meandering stubs are strategically added to the top right corner of the radiating structure. This addition leads to a significant decrease in the reflection coefficient, achieving |S_11_| levels as low as −30 dB at 5.8 GHz. For further improvement in the design, an extensive parametric analysis is employed. In step 3, the meandered stub line is incremented further, reaching toward the other end of the U-shaped element. Table 1 shows the single antenna's optimized parameters.

**Table 1:** Dimensions of the single-element antenna.

Parameters	Value (mm)	Parameters	Value (mm)	Parameters	Value (mm)
p_x_	15	p_y_	15	h	0.127
f	5.5	k_1_	4.5	w	1.75
f_1_	2	k_2_	6.25	b_1_	0.5
f_2_	0.5	k_3_	6.25	t	0.127
L	7	k_4_	4.5		
L_1_	8	b	0.5		

### 2.2 Step-by-step design of single element

**Fig. 2:**
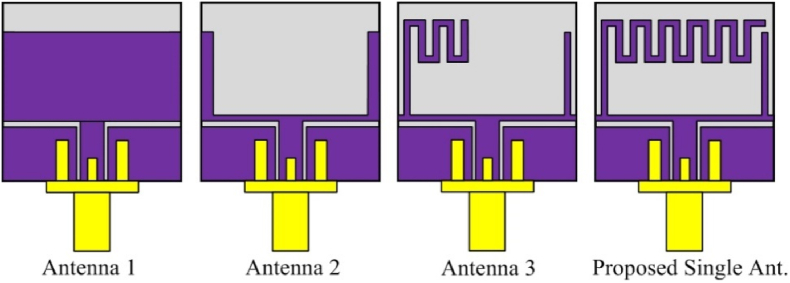
Design evolution of the proposed flexible single-unit antenna.

**Fig. 3:**
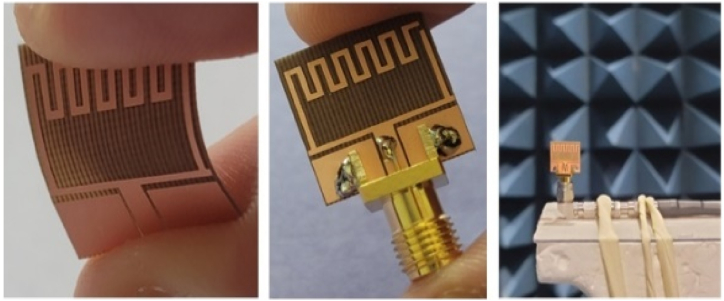
Fabricated prototype and measurement setup.

**Fig. 4:**
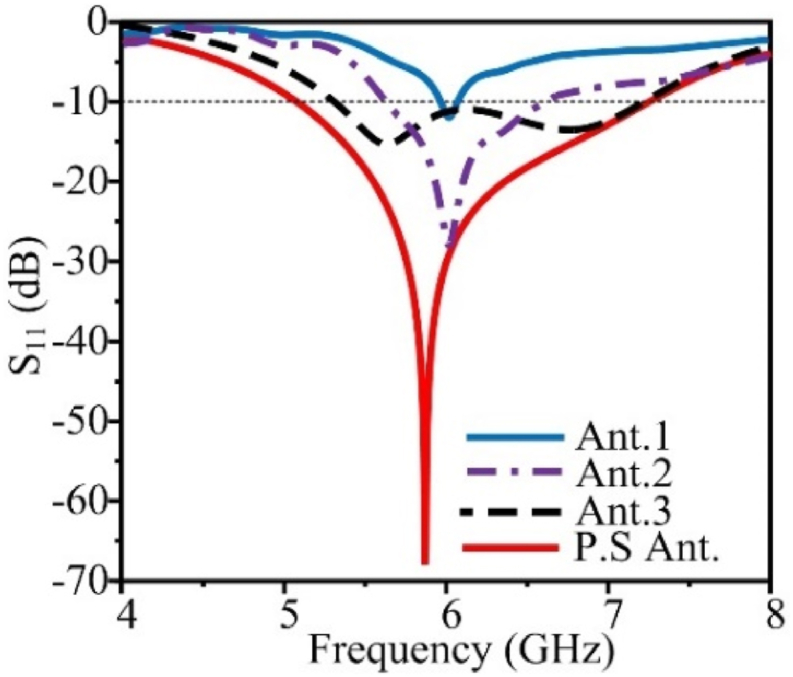
S_11_ parameters of different antenna design stages antenna–1 and antenna–2, antenna–3, and proposed single antenna.

The step–by–step design evolution of the proposed single–band antenna is illustrated in Fig. 2. The proposed single–band antenna evolved from the single–band coplanar monopole antenna (Antenna–1). The patch antenna offers a very limited bandwidth, therefore increasing the impedance bandwidth of the antenna. Antenna 1 is converted into a U-shaped radiator named Antenna 2. To improve the bandwidth further meandered lines are added at the upper left side of Antenna-2. The radiating patch of antenna-1 offers an operating frequency from 5.79 to 5.85 GHz. The horizontal line provides a wider resonance at 5.8 GHz, therefore more lines have been added to achieve the targeted bandwidth. The fabricated single-element antenna is shown in Fig. 3. While the step-by-step improvement in the bandwidth of the antenna is shown in Fig. 4.

The proposed design is validated through the fabrication and measurement of a prototype of the single antenna. The antenna is tested on a commercially available vector network analyzer (Agilent Technologies E8364B). The radiation characteristics measuring setup in the anechoic chamber are almost similar to the simulation results.

**Fig. 5:**
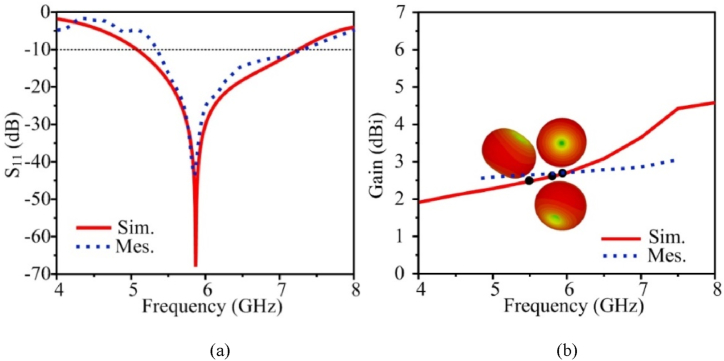
Measurement and simulated results of the proposed single element antenna (a) |S_11_| characteristic and (b) gain.

In Fig. 5(a) the |S_11_| for the single-element antenna simulation and measurement data is displayed. The simulated and measured results exhibit excellent correspondence. The −10 dB impedance bandwidth of the single element is from 5.06 to 7.24 GHz. The antenna in Fig. 5(b) shows the maximum stable gain of 4.63 dBi at 5.9 GHz. Due to measuring equipment losses, the measured value of gain is a little lower than the simulated values.

**Fig. 6:**
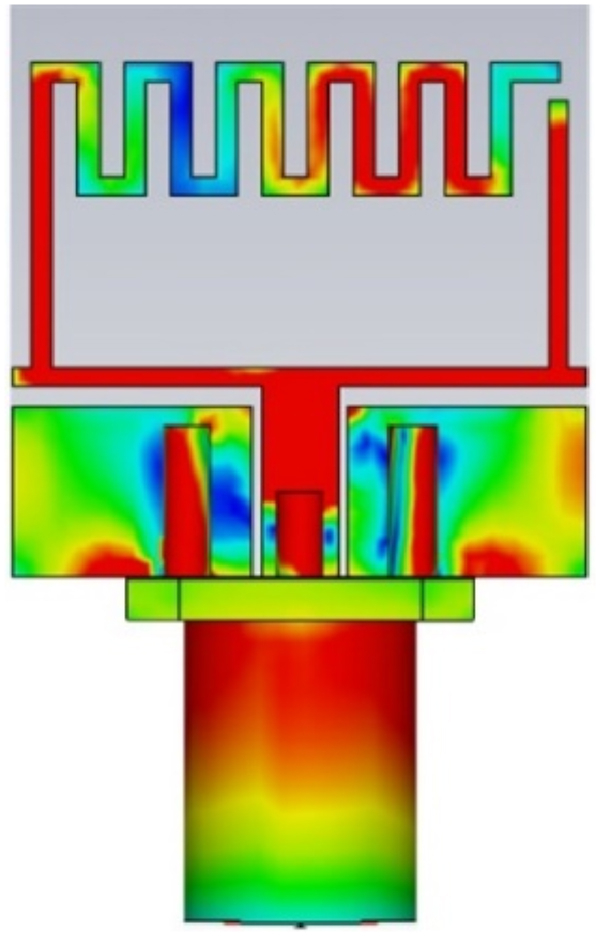
Current distribution of the proposed antenna at 5.8 GHz.

Fig. 6 shows the current distribution of the antenna for various operating frequencies. The active elements can be seen in the figure, which validates the design process. The U-shaped patch exhibits radiation within the 5.8 GHz frequency range. Furthermore, the open stub on the left provides a broader resonance band, covering 5.8 GHz (ranging from 5.53 GHz to 7.23 GHz). The current distribution reaches its maximum at the U-shaped segment of the radiating element. A comparable current distribution magnitude is observed at the far-right extremity of the meandered stub within the individual element. The presence of the meandered patch has a substantial impact on the overall antenna characteristics, as evident from the current distributions across the antenna structure.

## 3 MIMO antenna design and mutual coupling reduction

**Fig. 7:**
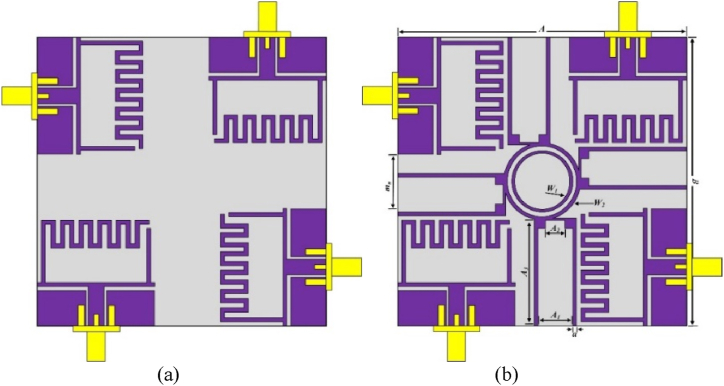
MIMO antenna configuration, (a) without and (b) with decoupling structures.

The MIMO antenna configuration is realized by replicating the single element and inserting a parasitic patch. The MIMO is designed by cloning the single unit into four units by translating the units perpendicularly in the *z*-axis to achieve conformal adaptation. The configuration of the MIMO antenna is dipicted in Fig. 7(a) without decoupling structure and 7(b) with proposed decoupling structure.

This configuration enables the antenna system to receive and transmit signals with different polarization orientations, which improves performance in a variety of propagation scenarios. The decoupling structure is formed by the combination of a pair of circular rings loaded with 4-open-ended U-shaped stubs to improve the isolation characteristics of the MIMO design. Initially, U-shaped stubs are loaded, and the spacing between the stubs and MIMO elements is optimized to achieve the best possible results. Afterwards, stubs are connected using a rectangular loop, however, due to the sharp edges of the rectangular loop the impedance matching gets disturbed. Thus, instead of a rectangular loop a circular loop is loaded which helps to achieve good impedance matching at the targeted band along with low mutual coupling. The proposed flexible MIMO antenna offers bandwidth from 5.37 to 7.34 GHz with a maximum gain of 4.63 dBi using omnidirectional stable radiation pattern characteristics that satisfy the need for 5.9 GHz V2X communication.

**Fig. 8:**
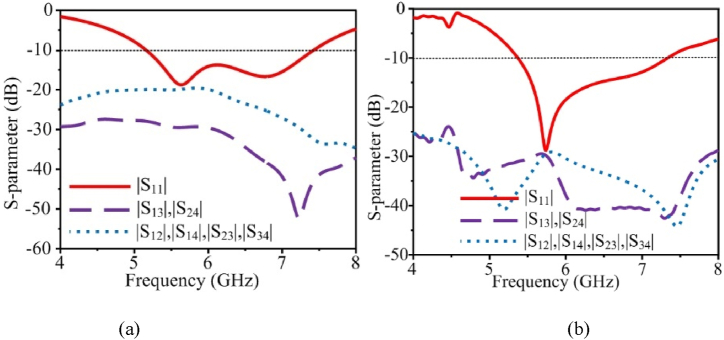
Transmission coefficient for MIMO antenna, (a) without decoupling structure, (b) with decoupling structure.

A 4-port scheme is utilized to design the MIMO structure. The S-parameter outcomes of the MIMO antenna are illustrated in Fig. 8(a) with and without decoupling structure. From Fig. 8(b) it can be seen that, without any decoupling mechanism, optimum perpendicular isolation values of (|S_13_| and |S_24_|) and for the adjacent antennas (|S_12_|, |S_14_|, |S_23_|, and |S_34_|) is 20 dB, however, by applying the decoupling structure, this value is increased to 29 dB. The additional parameter after applying the decoupling structure is shown in Table II.

**Table 2:** Additional parameters used for MIMO design.

MIMO antenna with decoupling structure parameters			
Parameters	Value (mm)	Parameters	Value (mm)
A	37	B	37
A_1_	4.4	a	0.5
A_2_	2.5	w_1_	0.5
A_3_	13.8	w_2_	0.5
m_n_	7		

**Fig. 9:**
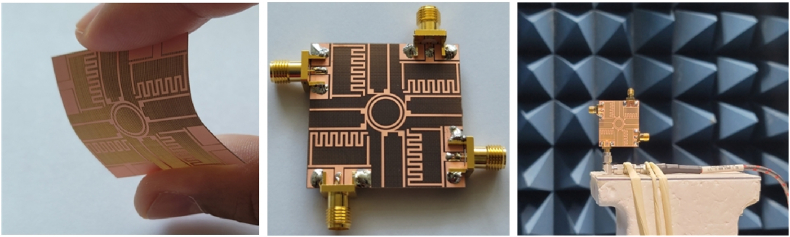
Fabricated prototype and far-field measurements setup for the flexible MIMO antenna.

The transmission coefficient in Fig. 9 illustrates the substantial mutual coupling between antenna parts offered by this antenna. The curves for the transmission coefficient for the nearby antennas |S_12_|, |S_14_|, |S_23_|, and S_34_ are the same. The |S_13_| and |S_24_| curves are identical, as well as the antennas that are perpendicular to each other. The MIMO performances and data rate are both lowered by the MIMO antenna elements' mutual coupling. For typical communications, a 20 dB isolation level might be sufficient. However, for WLAN communication systems, it's crucial to have constant connectivity and a fast data rate. The proposed design has |S_12_|, |S_14_|, |S_23_|, and |S_34_| minimum value of −29 dB throughout the operational region. In addition, the diagonal elements have minimum transmission coefficient values of |S_13_| and |S_24_| of −28 dB throughout the operational region.

## 4 Results and discussion

A prototype of the suggested MIMO antenna is made and measured to assess the proposed design. In Fig. 9. The manufactured antenna image and associated measuring set-up demonstrate the prototype antenna is used as a receiver, and a well-calibrated normal gain horn antenna is used as a transmit antenna. To deliver consistent power reception, amplifiers are employed. The antenna is rotated during testing to measure the radiation intensity at various angles. The diversity performance from the viewpoints of the transmission coefficient, ECC, diversity gain, mean effective gain (MEG), and channel capacity loss (CCL) is examined to assess the reliability of the proposed MIMO antenna system.

### 4.1 Reflection coefficient, transmission coefficient, and radiation pattern

**Fig. 10:**
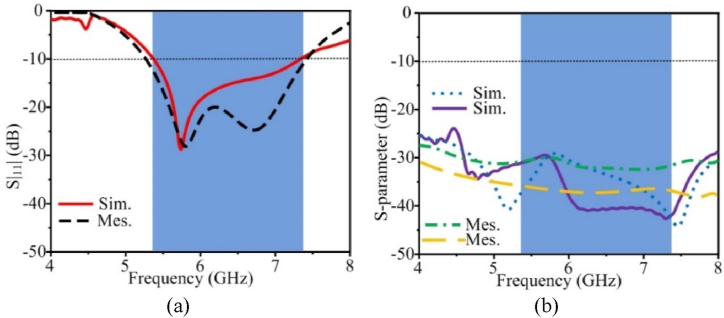
Simulation and measurement findings for the flexible MIMO antenna, (a) transmission coefficient and (b) reflection coefficient.

**Fig. 11:**
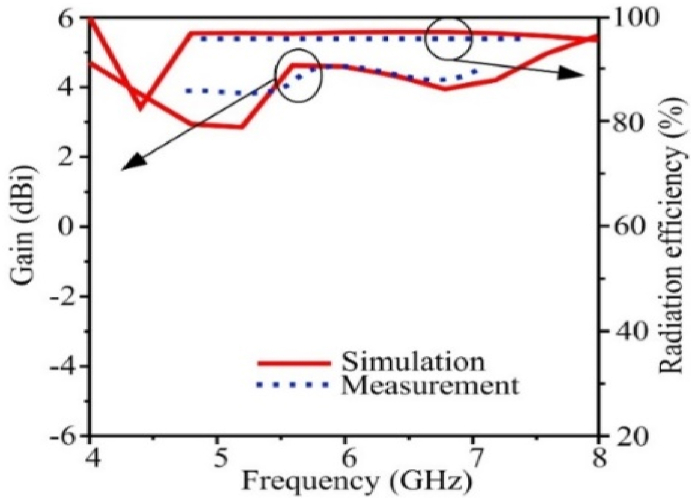
Gain and radiation efficiency of the proposed flexible MIMO antenna.

In Fig. 10(a), reflection coefficient curves for the proposed MIMO antenna are depicted using simulation and measurement data. To reduce mutual coupling, by using the suggested hybrid structure between the MIMO components, MIMO antennas have a somewhat different |S_11_| 10 dB bandwidth than single-element antennas. The constructed prototype has an impedance bandwidth of 5.53–7.23 GHz, which is within 10 dB. The results of the simulation and measurement are highly congruent. The transmission coefficient represents the interdependence of the MIMO components. Fig. 10(b) shows the measured and simulated transmission coefficients for the proposed MIMO antenna. The mutual coupling between the antennas is quite low sections when implementing the suggested integrated decoupling arrangement. The highest mutual coupling of the antennas is 29 dB, while the minimum mutual coupling is 43 dB over the entire working bandwidth. Fig. 11 shows the single element's gain and radiation efficiency based on simulation and measurement data. At the whole operational frequency, the antenna's highest stable gain is 4.63 dBi, and its radiation effectiveness is over 98 %. Due to measurement equipment losses, values for gain and radiation efficiency that were measured were slightly lower than those that were simulated.

**Fig. 12:**
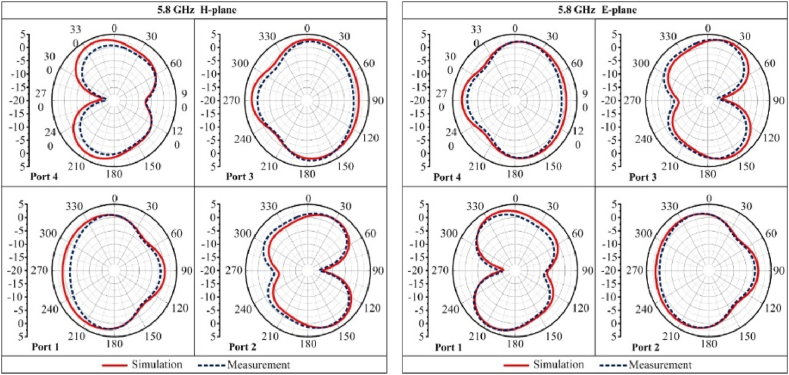
Radiation patterns of the proposed flexible MIMO antenna.

The simulated and measured radiation patterns of the proposed antenna are compared in the *E*- and *H*-radiating planes at 5.8 GHz. Fig. 12 shows the proposed MIMO antenna's polar plot radiation characteristics. At the operating frequencies, the proposed MIMO antenna delivers an omnidirectional radiation pattern.

### 4.2 Diversity gain

**Fig. 13:**
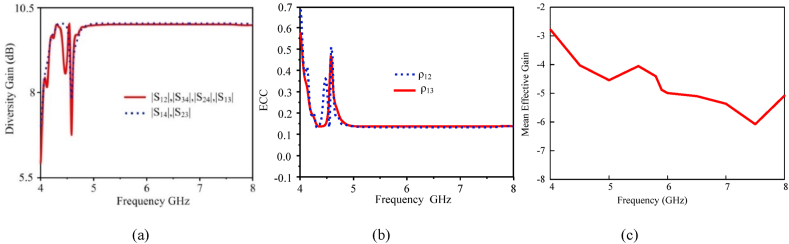
Performance metrics of proposed flexible MIMO antenna: (a) Diversity gain (b) envelope correlation coefficient and (c) MEG.

The diversity gain (DG) of the MIMO antenna system is a measure of the improvement in signal reliability achieved by exploiting multiple antennas. It characterizes the reduction in error rates, fading effects, and signal degradation when transmitting and receiving signals in complex and fading-prone environments. A higher diversity gain indicates improved system performance in terms of error rate reduction and signal robustness. Equation (6) [31,32] is used to calculate the DG for the proposed MIMO antenna, which is displayed in Fig. 13(a). A diversity gain of more than 10 dB is provided by the proposed MIMO antenna for each antenna element, a value that is practically identical to the optimum value.

(6) $$DG=\sqrt[{10}]{1-|\rho ij}|$$

### 4.3 Envelope correlation coefficient mean effective gain and Tarque of the MIMO system

In MIMO antenna systems, maintaining a low envelope correlation coefficient (ECC) is essential to harness the full advantages of multiple antennas, such as spatial diversity, reduced fading, and enhanced signal quality. The reduction in envelope correlation is often achieved through careful antenna design, positioning, and signal processing techniques. The ideal value for ECC is zero, indicating complete uncorrelation between received signals, but in practical scenarios, a value less than 0.5 is considered acceptable. In the case of the proposed MIMO system, the calculated ECC is exceptionally low, measuring less than 0.001. Equation (7) can be utilized to compute the ECC for the MIMO system's transmission coefficient and far-field radiation patterns, respectively [33,34]. These equations provide a quantitative assessment of the correlation between the envelopes of received signals, which is a critical factor in evaluating the performance of MIMO systems. A low ECC value signifies that the signals received by different antennas are nearly independent in magnitude, which is favorable for optimal MIMO performance. The ECC of the antenna is shown in Fig. 13(b).

(7) $$\rho_{eij}=\frac{{\left|\iint_{0}^{4\pi }\left[\vec{R_{i}}\left(\theta ,\varphi \right)\times \vec{R_{j}}\left(\theta ,\varphi \right)\right]d\Omega \right|}^{2}}{\iint_{0}^{4\pi }{\left|\vec{R_{i}}\left(\theta ,\varphi \right)\right|}^{2}d\Omega \iint_{0}^{4\pi }{\left|\vec{R_{j}}\left(\theta ,\varphi \right)\right|}^{2}d\Omega }$$

In a multipath environment, an antenna's ability to receive electromagnetic power is measured by Mean Effective Gain. It is the mean received power divided by the antenna's mean incident power. Fig. 13 c shows the value of MEG. The chart shows that the antenna value falls within the suitable MIMO system range of less than −3 dB. To determine this, use equation (8)

(8) $${MEGi}_{=0.5}\left(1-\;\sum_{i=1}^{N}\left|Sij\right|\right)$$

Where i is the port under observation and n is the number of antennas.

### 4.4 Surface current

**Fig. 14:**
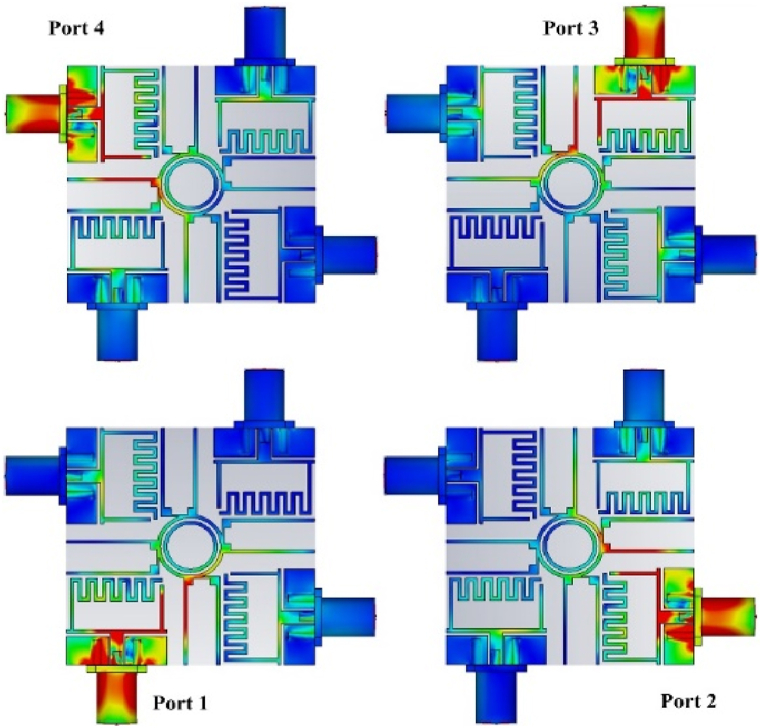
Surface current distribution of proposed flexible MIMO antenna of the different port extensions at 5.8 (GHz).

Fig. 14 depicts various stimulating points of the surface current distribution for the proposed MIMO antenna at 5.9 GHz. When port-1 is operational in the absence of a decoupling device, a considerable current flows from the stimulated patch across the adjacent MIMO antenna components, resulting in a high level of mutual coupling. In contrast, the proposed decoupling technique significantly lowers mutual coupling across the various components of the MIMO antenna, resulting in high isolation. This prevents a significant current from propagating to other locations. The surface current distribution at stimulated port-3 confirms this effect, demonstrating that the proposed decoupling structure successfully mitigates mutual coupling among the MIMO antenna components, improving antenna performance.

### 4.5 Link budget

A communication signal is transmitted from a transmitter to a receiver through a communication medium, such as a cable, radio waves, optical fiber, or waveguide. A link budget calculates a communication link's performance while considering all the power, gains, and losses a signal in a communication system encounters. The proposed MIMO antenna's link budget is calculated using Equation (8) given below to see if it is suitable for V2X transmission [35].

(9) $$P_{rx}=P_{tx}+G_{tx}-L_{tx}-L_{fsp}+L_{m}+G_{rx}-L_{rx}$$

Where the transmit power is denoted by *Prx* and the received power is represented by *Ptx* is the transmit power in dBm, *Ptx* is the received power in dBm, *Gtx* is the antenna gain in dBi at the transmitter, *Ltx* is the total system loss in dB at the transmitter, *Lfsp* is the total propagation losses in dB between the transmit and receive antennas, *Lm* is miscellaneous losses (fade margin, polarization misalignment, etc.) in dB, *Grx* is the antenna gain in dBi at the receiver and, *Lrx* is the total system loss in dB at the receiver. For a system that has been specifically constructed, only the loss of free space path ($L_{fsp}$) is dependent on both frequency (f) and distance (d). The other variables stay the same. Equation (9) is used for calculating the free space path loss ($L_{fsp}$) [36].

(10) $$L_{fsp\left(\frac{dB}{km}\right)}=32.4+20\;\log_{10}\left(d_{km}\right)+20\;\log_{10}\left(f_{MHz}\right)$$

**Fig. 15:**
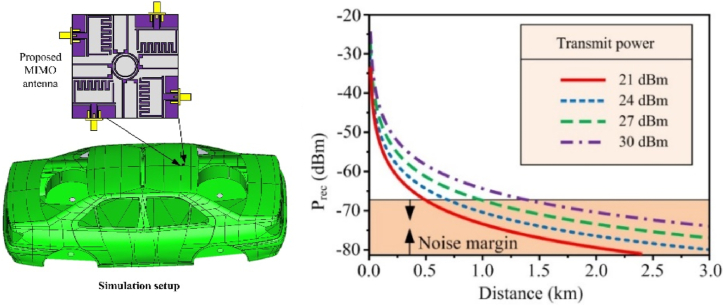
The link budget for the proposed flexible MIMO antenna at various transmits powers.

On is entered into MATLAB as a tool to compute the link budget of the proposed MIMO antenna. *Grx* = *Gtx* = 4.63 dBi, *Ltx* = surge kit (0.5) + cable (1.7) + connectors (0.5) + mismatch (0.511) ≈ 3.2 dB, *Lm* = 0.5 dBm, *Lrx* = surge kit (0.5) + cable (0.85) + connectors (0.5) + mismatch (0.511) ≈ 2.35 dB, the link budget outcome is displayed in Fig. 15. The following equation (10) is used to determine the antenna's sensitivity or minimum detectable signal [17].

(11) $$MDS=10\;\log_{10}\left(\frac{kt}{1mw}\right)+Noise\;Figure+10\;\log_{10}\left(Bandwidth\right)$$

In this equation (8), MDS stands for minimum detectable signal, T is the temperature (290 k), and k is the Boltzmann constant (228 dBW/(KHz)). The suggested antenna will have a minimum sensitivity of −67.25 dBm at a channel bandwidth of 1970 MHz and a noise figure of 13.8 dB.

The WHO (World Health Organization) and the FCC both recommend using a maximum of 1 W of antenna power in metropolitan areas. The antenna testing for the V2X communication system should also use 21 dBm of transmit power, as recommended by 5GAA, to guarantee the antenna's performance in real-world applications [37]. As illustrated in Fig. 15, the link budget for the proposed MIMO antenna is estimated for transmit powers ranging from 126 mW to 1W. The link budget graph shows that the antenna can communicate with one another across 1.5 km away with a minimum transmit Tx power of 21 dBm, but 5G requires a minimum distance for communication of 300 m between the antennas.

## 5 Comparative analysis of performance with prior research

The performance comparison of the proposed antenna with related works is summarized to locate this work in the state of the art in Table 3. The comparison involves the antenna's electrical size, isolation, IBW, radiation efficiency (RE), gain ECC, and flexibility. The proposed antenna has the additional benefit of flexibility other than having a compact size and moderate gain. Flexibility in V2X communication antennas ensures that they can meet the diverse and dynamic requirements of vehicular communication, accommodating different vehicle types, environmental conditions, and user preferences. This adaptability is vital for reliable and efficient V2X communication systems.

**Table 3:** Comparison with the state of the art.

Reference	Antenna Size ((λ^3^))	Isolation (dB)	IBW	RE (%)	Gain (dBi)	Flexible	ECC
[14]	1.084 × 1.084 × 0.0017 (4 element)	>22	2.9–10.86 (GHz)	97 %	4	No	<0.01
[17]	1.2384 × 1.234 × 0.025 (4 element)	>34	5.83–5.94 (GHz)	94 %	7.68	No	<0.001
[21]	0.75 × 0.63 × 0.025 (4 element)	>13	4.98–5.9 (GHz)	–	4	yes	<0.04
[23]	0.63 × 0.63 × 0.025 (4 element)	>13	5.3–6.7 (GHz)	79 %	5.2	No	<0.001
[24]	0.433 × 0.433 × 0.0133 (4 element)	>15.4	5.6–5.8 (GHz)	72 %	1.41	No	<0.01
[25]	1.212 × 0.611 × 0.0266 (2 element)	>13	5.4–5.95 (GHz)	–	3.74	No	<0.001
[26]	1.006 × 0.667 × 0.0266 (2 element)	>17	5.15–6.12 (GHz)	–	4.5	No	<0.3
[27]	1.66 × 0.833 × 0.0133 (2 element)	>12	5.1–6 (GHz)	75 %	6.57	No	<0.5
[28]	0.833 × 1.33 × 0.0126 (2 element)	>15	4.18–6.58 (GHz)	80 %	6	No	–
[30]	0.917 × 1.334 × 0.025 (2 element)	>29	5.1–5.6 (GHz)	–	8	No	<0.01
[32]	1.00 × 1.451 × 0.0100 (2 element)	>29	5.85–5.95 (GHz)	95 %	8.3	Yes	<0.001
**Prop. Antenna**	0.617 × 0.617 × 0.0021 **(4 elements)**	**>29**	**5.53**–**7.32 (GHz)**	**98 %**	**4.63**	**Yes**	**<0.001**

## 6 Conclusion

This article proposes a conformal, compact, and geometrically straightforward monopole antenna together with its 4-port Multiple-Input-Multiple-Output (MIMO) configuration for V2X communications. A combination of a pair of circular rings loaded with 4-open-ended U-shaped stubs to improve the isolation between the elements of the MIMO system. Finally, to enhance the impedance matching across all bands, the antenna is adjusted using comprehensive parametric analysis. From 5.06 to 7.24 GHz, the single element antenna exhibits |S_11_| −10 dB magnitude, reaching a peak low magnitude of −68 dB. The suggested flexible MIMO antenna features omnidirectional stable radiation pattern characteristics, a peak gain of 4.63 dBi, and a bandwidth from 5.37 to 7.34 GHz. To verify the findings, the MIMO configuration of the antenna prototype is etched on a 37 × 37 × 0.127 mm3 Rogers 5880 laminate board. The MIMO antenna demonstrated is ideal for V2X and has a low envelope correlation coefficient (ECC), good capacity loss, and strong diversity gain.

## Funding

This work was supported by the Institute of Information & Communications Technology Planning & Evaluation (10.13039/100019635IITP) grant funded by the Korean government (10.13039/501100014188MSIT) (No. 2022-0-01031, Development of measured 10.13039/100000863EMF big data analysis and management platform). Authors' contributions and conflict of interest: All authors of this research paper have directly participated in the planning, execution, or analysis of this study. The authors declare that they have no known competing financial interests or personal relationships that could have appeared to influence the work reported in this paper.

## Data availability

No data was used for the research described in the article.

## CRediT authorship contribution statement

**Uktam Fakhriddinovich Azimov:** Writing – original draft. **Anees Abbas:** Writing – review & editing. **Seong-Wook Park:** Conceptualization. **Niamat Hussain:** Conceptualization. **Nam Kim:** Project administration, Funding acquisition.

## Declaration of competing interest

The authors declare that they have no known competing financial interests or personal relationships that could have appeared to influence the work reported in this paper.
